# Vascular disrupting agents in clinical development

**DOI:** 10.1038/sj.bjc.6603694

**Published:** 2007-03-20

**Authors:** P Hinnen, F A L M Eskens

**Affiliations:** 1 Department of Medical Oncology, Erasmus University Medical Center, PO Box 2040, Rotterdam 3000 CA, The Netherlands

**Keywords:** vascular disrupting drugs, clinical trials, angiogenesis inhibitors

## Abstract

Growth of human tumours depends on the supply of oxygen and nutrients via the surrounding vasculature. Therefore tumour vasculature is an attractive target for anticancer therapy. Apart from angiogenesis inhibitors that compromise the formation of new blood vessels, a second class of specific anticancer drugs has been developed. These so-called vascular disrupting agents (VDAs) target the established tumour vasculature and cause an acute and pronounced shutdown of blood vessels resulting in an almost complete stop of blood flow, ultimately leading to selective tumour necrosis. As a number of VDAs are now being tested in clinical studies, we will discuss their mechanism of action and the results obtained in preclinical studies. Also data from clinical studies will be reviewed and some considerations with regard to the future development are given.

Tumour-related angiogenesis is essential for tumour growth and metastases formation. It is a complex process in which vascular endothelial growth factor (VEGF) produced by tumour cells plays a predominant role (Ferrara *et al*, 2003). Binding of VEGF to the transmembrane endothelial VEGF tyrosine kinase receptors (VEGFR) type 1 or 2 initiates a cascade of intracellular signaling pathways resulting in endothelial cell proliferation and the formation of new blood vessels. Apart from VEGF, basic fibroblast growth factor, platelet-derived growth factor, interleukin-8 and insuline-like growth factor are proangiogenic factors. Natural antiangiogenic factors produced by tumour and host cells are tumour necrosis factor alpha (TNF-*α*), serotonin (5-HT), nitric oxide (NO), thrombospondin, angiostatin and endostatin.

Inhibiting angiogenesis has become a challenge in the development of a totally new class of anticancer drugs as was already acknowledged in 1971 by Folkman (1971). Angiogenesis inhibitors can be divided into two groups, monoclonal antibodies (Moabs) and small molecule tyrosine kinase inhibitors (TKIs). Bevacizumab is a humanised Moab targeting VEGF, which has shown clinical activity in combination with cytotoxic chemotherapy in metastatic colorectal cancer, non-small cell lung cancer (NSCLC) and breast cancer (Hurwitz *et al*, 2004; Ramaswamy *et al*, 2006; Sandler *et al*, 2006). As single agent bevacizumab has demonstrated acivity in metastatic renal cell carcinoma (Yang *et al*, 2003).

Apart from Moabs, a large number of small-molecule VEGFR TKIs have been explored in clinical studies. Results in randomised studies in renal cell carcinoma with the broadspectrum TKIs sunitinib and sorafenib have resulted in their regulatory approval for this disease (Escudier *et al* 2007; Motzer *et al*, 2007). Most other TKIs so far have either only been tested in smaller phase I and II studies, or have failed to show meaningful effects in larger randomised phase III studies (Koehne *et al*, 2006). Theoretically, it is conceivable that angiogenesis inhibitors will exert optimal activity in a situation of minimal residual disease with high-angiogenic potency such as could be the case in the adjuvant setting.

Although inhibiting angiogenesis thus seems to be successful in various conditions, an urgent need for more optimal treatment options in metastatic disease exists.

For example, what to do with the already established tumour-related vasculature?

Vascular targeting strategies can be divided into two different approaches: as mentioned above an antiangiogenic approach, but apart from that a so-called vascular disrupting approach has emerged (Siemann *et al*, 2005). Vascular disrupting agents (VDAs) target endothelial cells and pericytes of the already established tumour vasculature. Although this approach looks very interesting from a theoretical point of view, one of the critical issues one could raise is that of tumour specificity; do VDAs selectively target tumour-related endothelium or is there a more general vascular targeting effect with a risk of subsequent ischaemic complications? In the following sections we will discuss in more detail the hypothesised mechanisms of action of VDAs and will review results of preclinical and clinical studies performed so far. Finally, we will give some thoughts on were to go with VDAs in future studies.

## VDAS AND THEIR TARGET

Indirect killing of tumours by compromising their vascularisation is a potentially attractive anticancer treatment approach. On the one hand drug resistance is not likely to appear because the targeted endothelial cell has much greater genetic stability than neoplastic cells (Kerbel and Folkman, 2002). Also drug delivery is likely to be without compromise, as the endothelium of the tumour vasculature is easily accessible. Lastly, and theoretically, vascular shutdown is likely to result in a massive ‘downstream’ tumour cell killing.

**Figure 1:**
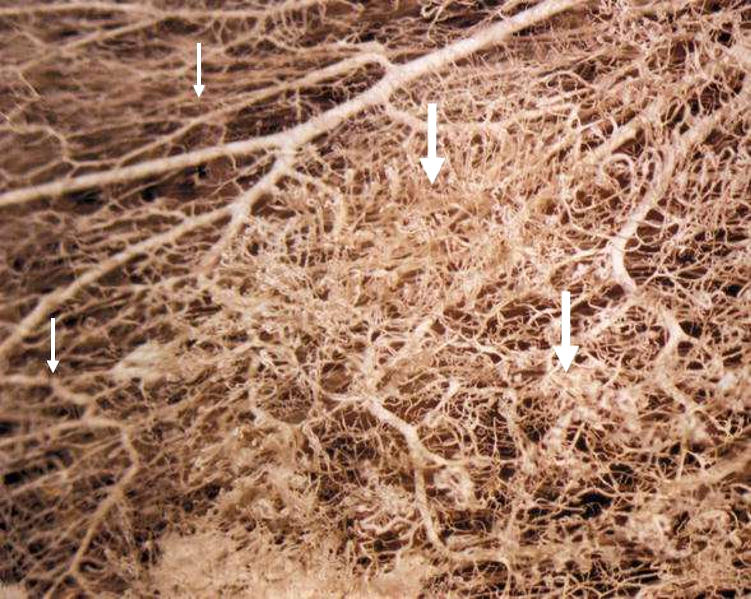
Architectural difference between vasculature in normal (small arrows) and tumour tissue (thick arrows).

Selective vascular shutdown suggests a structural difference in endothelium of tumour vessels compared to that of normal vessels. Indeed, tumour vasculature is, among others, marked by a high rate of endothelial cell proliferation, the absence of pericytes, abnormalities in the basement membrane and often an increased vascular permeability. Structurally, disorganised, tortuous, thin-walled vessels are seen that lack smooth muscle and pericyte coats and innervation (Figure 1) (Kakolyris *et al*, 2000; Konerding *et al*, 2001). Blood flow frequently is sluggish and at times might be stationary or even in a reversed direction (Tozer *et al*, 1990). Vessel diameters are irregular and lengths between branches are long, resulting in a high resistance to blood flow. A small decrease in perfusion pressure, which has little effect in normal tissue, therefore can be catastrophic to tumours.

Finally, endothelial cells are highly dependent on tubulin cytoskeleton for their motility, invasion, attachment, alignment and proliferation (Denekamp, 1982).

Most VDAs induce changes in endothelial cell shape by disruption of the cytoskeleton and cell-to-cell junctions. This results in increased permeability to proteins and an increased interstitial fluid pressure, which might be sufficient to reduce vessel diameter. Plasma leakage also leads to increased blood viscosity resulting in decreased blood flow and roulaux formation. Another factor contributing to the vascular shutdown is the activation of platelets through contact with basement membrane components, which are exposed. All together this cascade of events results in vascular shutdown more selectively in tumour endothelium than normal endothelium. As stated previously, it is suggested that the inhibition of blood flow and the subsequent compromised supply of oxygen and nutrients will induce necrosis of many tumour cells downstream.

**Figure 2:**
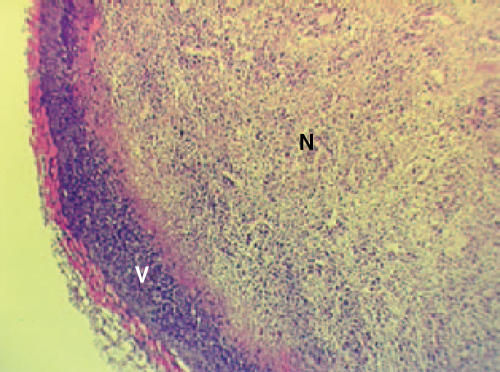
Typical example of a tumour with a viable rim (V) and central necrosis (N) following exposure to a vascular disrupting agent.

Compared to the antiangiogenic approach of both TKI and Moab, the vascular disrupting approach therefore seems to be cytotoxic rather than cytostatic. However, in preclinical models it has been observed that following exposure to a VDA, only the centre of a tumour becomes necrotic, with a viable rim (Figure 2) remaining in the periphery. This rim of viable tumour cells presumably survives because it derives nutritional support (most likely via diffusion) from adjacent normal blood vessels that are typically less responsive to VDAs.

To demonstrate biological activity of VDAs in preclinical and clinical studies, noninvasive techniques such as dynamic contrast-enhanced magnetic resonance imaging (DCE–MRI) and positron emission tomography scans (PET) have been explored. This type of imaging can demonstrate changes in tumour perfusion and tumour viability (Beauregard *et al*, 1998; Galbraith *et al*, 2003). Although data to date look very promising, one must realise that both techniques are not yet validated to predict antitumour activity or real patient benefit in the clinical situation.

## VDAS IN CLINICAL DEVELOPMENT

Vascular disrupting agents have been divided into two types, small molecule and ligand directed VDAs. We will focus on small molecule VDAs because they are in a more advanced stage of clinical development. Small molecule VDAs can be divided into two groups; the tubulin-binding agents and the flavonoids. Their mechanism of action is somewhat different as will be discussed. Tubulin-binding agents are combretastatin, AVE8062, ZD6126, ABT-571, MN-029 and the dolatastatin derivative TZT-1027. Of the flavonoids only 5,6-dimethylxanthenone-4-acetic acid (DMXAA) will be discussed.

## I. TUBULIN-BINDING AGENTS

These agents work by acting at the colchicines-binding site of the *β*-subunit of endothelial tubulin, resulting in depolymerisation of microtubules and disorganisation of actin and tubulin. Disruption of the endothelial cytoskeleton results in conformational changes leading to loss of blood flow. In addition to this, a recent study showed that the typical microtubule-destabilising agent combretastatin A4 phosphate (CA4P) also selectively disrupts the VE-cadherin/*β*-catenin complex interfering with cell–cell contact (Vincent *et al*, 2005). Loss of this cell–cell contact increases vascular permeability leading to increased interstitial pressure and additional loss of blood flow. In addition to these effects, the already mentioned loss of cell–cell contact results in the exposure of the already often abnormal basement membrane, which in turn can result in the induction of the coagulation cascade with subsequent thrombus formation. Tumour-related endothelial cells are much more sensitive to the activity of tubulin-binding agents than normal endothelial cells (Chaplin and Dougherty, 1999).

### Combretastatin A4 phosphate

Combretastatin A4 phosphate is a water-soluble prodrug of combretastatin A4 (CA4). Following administration, CA4P is rapidly cleaved to CA4 and binds tubulin at or close to the colchicines-binding site (McGown and Fox, 1989). One of the first *in vivo* studies showed rapid, extensive and irreversible vascular shutdown and haemorrhaghic necrosis following a single dose of CA4P. A pronounced and sustained reduction in functional vascular volume was observed following drug administration at a dose much lower than the maximum-tolerated dose (MTD) (Dark *et al*, 1997). Histological as well as DCE–MRI studies in preclinical models show that the antivascular effects of CA4 are restricted to the core of the tumour, leaving viable tumour cells at the periphery (Beauregard *et al*, 1998). Combretastatin A4 shows different activity in normal and tumour endothelium in preclinical models; Tozer *et al* (1999) showed a 100-fold decrease in blood flow in p22 carcinosarcomas with a much smaller reduction in blood flow in the spleen, skeletal muscle and brain. No significant reduction in blood flow was seen in heart, kidney and intestine.

Three phase-I trials of CA4P in humans have been published (Table 1). In the first study by Rustin *et al* (2003a) CA4P was given weekly for 3 weeks followed by a week gap . Thirty-four patients with advanced solid tumours received 167 infusions. Up to 40 mg m^−2^, the only drug-related toxicity was tumour pain in 35%. Tumour pain was not considered a dose-limiting toxicity (DLT) because it could be controlled by analgesics. Tumour viability and tumour blood flow were assessed by PET and DCE–MRI (Galbraith *et al*, 2003). Dose-limiting toxicity were fatal ischaemia in previously irradiated bowel, vasovagal syncope, motor neuropathy and reversible ataxia. Other side-effects were hypertension (35%), hypotension (30%), tachycardia (53%), bradycardia (24%), nausea (21%), fatigue (23%), visual disturbance (9%) and dyspnoea (6%). The drug was generally well tolerated and no myelosuppression, alopecia and mucositis were seen. One partial response was seen (metastatic adrenocortical carcinoma). The recommended phase-II dose of 52–68 mg m^−2^ was based upon clinical tolerability and the assessment of biological activity by means of PET and DCE–MRI analysis.

In a second phase-I study, Stevenson *et al* (2003) used a daily infusion for 5 days every 3 weeks . Thirty-seven patients received 133 cycles. Dose-limiting toxicities were tumour pain, reversible sensorimotor neuropathy, syncope and dyspnoea. No cardiotoxicity or electrocardiographic changes were seen. One patient with metastatic sarcoma had a partial response, and 14 patients showed stable disease. The recommended phase-II dose was 52 mg m^−2^.

Dowlati *et al* (2002) used a once every 3 weeks schedule. Twenty-five patients received 107 cycles. Dose-limiting toxicities were cardiac ischaemia and dyspnoea in two patients with pre-existing cardiovascular disease. A significant decline in gradient peak tumour blood flow by DCE–MRI was observed in six patients treated at 60 mg m^−2^. One complete response was observed in a patient with anaplastic thyroid cancer, whereas two patients experienced freedom from disease progression lasting more than 12 months. Dosages up to 60 mg m^−2^ as a 10-min infusion defined the upper boundary of the MTD. Cooney *et al* (2004) determined the cardiovascular safety profile of CA4P in the same patient cohort. They observed asymptomatic QTc prolongation as DLT. Apart from this, two patients had an acute coronary syndrome within 24 h after the infusion of CA4P .

All mentioned studies used a different dosing schedule (weekly, 3-weekly, daily for 5 days every 3 weeks) and showed that CA4P was safe, well tolerated and lacking haematologic toxicity. In all studies MTDs of 50–60 mg m^−2^ were set with consistent indications of antivascular effects observed by either DCE–MRI or PET. Currently CA4P is further explored as single agent in phase-II studies in patients with advanced anaplastic thyroid cancer.

Apart from single-agent approaches, CA4P has been studied in combination with carboplatin. Combretastatin A4 was given 3-weekly (27–36 mg m^−2^) 60 min after carboplatin (AUC 4–5). Dose-limiting toxicity was trombocytopenia (Bilenker *et al*, 2005).

In another ongoing study, induction chemotherapy using doxorubicin and cisplatin is followed by CA4P and radiation therapy in patients with newly diagnosed advanced anaplastic thyroid cancer.

Finally, CA4P is currently being explored in combination with carboplatin and paclitaxel in patients with advanced solid tumours.

### AVE8062

AVE8062 is a water-soluble analogue of CA4 with markedly enhanced antitumour effects. Preclinical studies have shown rapid and irreversible vascular shutdown in various different orthotopic tumour models. Complete stasis of blood flow was observed after 30 min, whereas blood flow in normal tissues was compromised but returned to pretreatment levels within 24 h. Tumour cell proliferation in different models was suppressed after drug infusion (Hori *et al*, 2002). So far only one phase-I single-agent study has been published in which nine patients received 48 infusions of AVE8062 (Table 1) (Tolcher *et al*, 2003). Cardiovascular effects consisting of asymptomatic systolic hypotension without elevation of CPK or troponin I levels or ECG changes were observed. Decreased tumour blood flow was observed by DCE–MRI at the 15.5 mg m^−2^ dose level. The half-life of AVE8062 was 15 min, but an active metabolite was formed with a half-life of 7 h. No response data are available. Currently, single-agent phase-I studies exploring other schedules of administration are ongoing.

As *in vivo* studies have shown synergistic activity of AVE8062 with oxaliplatin and docetaxel (Demers *et al*, 2006), clinical studies exploring these combinations are currently also ongoing.

### ZD6126

In preclinical models this agent demonstrated significant antitumour activity. Stasis of blood flow was seen at doses 1/8–1/16 of the MTD and occurred specifically in tumour tissue (Davis *et al*, 2002). Two phase-I studies, in which ZD6126 was given thrice weekly, have been presented (Gadgeel *et al*, 2002; LoRusso *et al*, 2002) (Table 1). One patient showed asymptomatic, reversible cardiac ischaemia with subsequent demonstration of coronary artery disease. Maximum-tolerated dose was set at 112 mg m^−2^, whereas biological activity indicated by a sustained decrease in tumour blood flow measured by DCE–MRI occurred at doses above 80 mg m^−2^. A third phase-I study has recently been published (Beerepoot *et al*, 2006). Here, ZD6126 was given weekly to 32 patients. Dose-limiting toxicity consisted of myocardial infarction and was observed at a dose of 10 mg m^−2^ in one patient. This patient was found to have a history of ischaemic heart disease. Two patients treated at 28 mg m^−2^ experienced DLT, one each with pulmonary embolus (disease-related?) and asymptomatic decrease in left ventricular ejection fraction. Maximum-tolerated dose was set at 20 mg m^−2^. In all three studies ZD6126 was well tolerated and only showed mild side effects such as anaemia, nausea, vomiting and constipation. So far no objective tumour responses have been observed. Currently, ZD6126 is being explored in metastatic renal-cell carcinoma.

### ABT-751

ABT-751 is a sulphonamide molecule that can be given orally, and has shown significant antitumour activity in a variety of tumour models (Ozawa *et al*, 2001). In a phase-I study, 39 patients with solid tumours were given ABT-751 once or twice daily for 7 days every 3 weeks (Table 1) (Hande *et al*, 2006). Dose-limiting toxicities were ileus and neuropathy at 300 mg daily. In the twice daily schedule, grade 3 ileus, constipation, abdominal pain and fatigue were observed. One minor response and four patients with stable disease lasting for 6 months were observed. The MTD and recommended phase-II doses for ABT-751 were 250 mg daily and 150 mg twice daily for 7 days every 3 weeks. Phase I/II studies are currently ongoing, evaluating the safety and efficacy of ABT-751 in combination with pemetrexed or docetaxel in patients with NSCLC.

### MN-029

One preclinical study was published using a rodent KHT sarcoma model. After intraperitoneal injection of 100 mg kg^−1^ a significant reduction in the functional vessel number was seen. Treatment with MN-029 resulted in dose-dependent tumour cell killing. Effects were enhanced by combining the agent with radiation and cisplatin chemotherapy (Shi and Siemann, 2005). Only one ongoing phase-I study has been reported so far (Table 1). In this study, 28 patients with various solid tumours recieved 110 cycles. Dose-limiting toxicity consisted of reversible cardiac ischaemia at 180 mg m^−2^. Seven patients had stable disease after 3 cycles. Dynamic contrast-enhanced magnetic resonance imaging analysis indicated significant dose-dependent reductions in tumour blood flow. Accrual at 225 mg m^−2^ continues (Ricart *et al*, 2006).

### TZT-1027

TZT-1027 is a synthetic derivative of dolastatin-10 with cytotoxic and antivascular activity. Three different treatment schedules have been explored in phase-I trials (Table 1). Schoffski *et al* (2004) performed a phase-I study in which 21 patients received TZT-1027 infusions at 3-weekly intervals. Dose-limiting toxicities were neutropenia, fatigue and short lasting peripheral neuropathy. Anorexia, alopecia and constipation were also seen. The recommended phase-II dose was set at 2.7 mg m^−2^. A second phase-I study, exploring day 1 and 8 every 3 weeks administration in 17 patients showed comparable DLTs as well as pain in the infusion arm lasting 1–2 days at a dose of 2.7 mg m^−2^ (DeJonge *et al*, 2005). Other side effects included nausea, fatigue, vomiting and diarrhoea. One patient with metastatic liposarcoma had an ongoing partial response for more than 54 weeks. The recommended dose for phase-II studies of TZT-1027 in this study was set at 2.4 mg m^−2^. A third phase-I study explored the combination of TZT-1027 with carboplatin in 14 patients (Greystoke *et al*, 2006). Dose-limiting toxicity consisted of neutropenia and grade 3 ileus. Other toxicities were comparable to those mentioned above. No pharmacokinetic interaction between carboplatin and TZT-1027 was observed. One patient with metastatic adenocarcinoma of the pancreas showed a partial response lasting 181 days. The recommended phase-II doses of TZT-1027 in combination with carboplatin AUC 5 was set at 1.6 mg m^−2^.

## II. FLAVONOIDS

### DMXAA

5,6-Dimethylxanthenone-4-acetic acid is an active analogue of flavone acetic acid causing DNA damage to endothelial cells that induces apoptosis in preclinical models (Corbett *et al*, 1986). In response to vascular damage 5-HT is released by platelets that further enhances the vascular effects (Baguley *et al*, 1997). Although the exact mechanism of action of DMXAA is unknown, its activity involves pathways leading to upregulation of the nuclear transcription factor Nf*κ*B, which leads to production of TNF-*α* and other cytokines (Ching *et al*, 2002). Tumour blood flow decreases and 5-HT levels increase. In addition, NO is produced in response to DMXAA, improving blood flow and vascular permeability, increasing the effects of TNF-*α* and 5-HT (Thomsen *et al*, 1991). How these forces oppose each other is unknown.

Two phase-I trials have been published so far (Table 1). Rustin *et al* (2003b) treated 46 patients with weekly infusions and documented rapidly reversible DLTs like urinary incontinence, visual disturbance (blurring, colour disturbance and photophobia) and anxiety. No tumour pain was seen. Maximum-tolerated dose was set at 3700 mg m^−2^. At dose levels of 650 mg m^−2^ and above a dose-dependent increase of 5-HT concentrations in plasma was seen. There was one unconfirmed partial response at 1300 mg m^−2^. In a second study, in which 63 patients received 3-weekly infusions, comparable DLTs were observed with additional confusion, slurred speech, tremor and possible left ventricular failure (Jameson *et al*, 2003). Asymptomatic transient QTc-prolongation was seen in 13 patients at high doses. One partial response was seen in a patient with cervical carcinoma. Maximum-tolerated dose was set at 3700 mg m^−2^.

Two randomised phase-II studies combining DMXAA with conventional chemotherapeutics have recently been published. Gabra, (2006) randomised 55 patients with recurrent ovarian cancer to receive paclitaxel (175 mg m^−2^), carboplatin (AUC 6) and DMXAA (1200 mg m^−2^). Preliminary data revealed no additional toxicity owing to the addition of DMXAA. Efficacy assessments are pending. In 78 patients with NSCLC, McKeage (2006) also found no additional toxicity when carboplatin and paclitaxel were combined with DMXAA. Initial response data suggest additional benefit from triple treatment compared to conventional therapy Currently, the efficacy and safety of DMXAA in combination with docetaxel is assessed in a phase-II study in patients with hormone refractory metastatic prostate cancer.

## FUTURE DEVELOPMENTS

Vascular disrupting agents are a new class of antivascular anticancer agents that are currently undergoing clinical studies. At this moment, mainly (ongoing) phase-I studies have been presented, although some compounds have already entered phase-II testing either as single agent or in combination with chemotherapeutics. Therefore, the real (added) value in terms of patient benefit cannot be fully assessed yet.

What distinguishes VDAs from other vascular targeting agents, how can we optimally assess their biological and clinical activity and how should these agents be taken forward?

When assessing the toxicity pattern observed so far in the various clinical studies described, it is obvious that with regard to mechanism of action tumour specificity is most likely to be of critical importance. Vascular disrupting agents disrupt the established abnormal tumour vasculature by targeting the immature dysmorphic endothelial cells. As mentioned earlier tumour endothelium is more vulnerable to the activity of VDAs, and therefore in the end selective tumour vascular shutdown is likely to occur. However, based upon the pattern of side effects observed in clinical studies, normal vascular endothelium seems to be affected by VDAs as well. Cardiac ischaemia and cardiac arrhythmias as well as reversible neurologic complications (the latter not with all agents) seem to underscore this issue and probably will remain dose limiting in future studies. Of critical importance therefore will be the assessment of biological activity at doses that can be administered safely. This will probably mean that in the design of early clinical studies the concept of looking for MTD will have to be replaced by the concept of looking for optimal biological dose, hereby assuming that the therapeutic window of these agents will permit us to do so. At this moment the probable optimal way to assess biologic or antivascular activity of VDAs is by repeated dynamic scanning, and therefore the role of DCE–MRI and PET analysis must be validated further. Vascular shutdown and decreased tumour blood flow as an indication of biological activity have meanwhile been demonstrated by DCE–MRI and/or PET analysis, whereas the occurrence of tumour pain following administration of several compounds also can be considered as an indication of biological and perhaps clinical activity. Whether biological activity will result in tumour size reduction and meaningful patient benefit has to be evaluated further in phase II and randomised phase-III studies. The assessment of cardiac and neurological complications that can occur in these studies necessitates optimal communication between oncologists and other specialists, and this will also be important when patients willing to be enrolled in studies are being screened (Heeckeren van, 2006). This is, as we think, a nice example of the tremendous practical consequences the introduction of a new class of anticancer drugs could and even should have.

When looking at the observed biological effects induced by VDAs, the induction of central tumour necrosis whereas leaving a viable rim at the periphery seems to be a consistent finding. Probably, this means that there is a rationale to combine VDAs with other treatment strategies. Many theoretical combinations can be thought of, and combining VDAs with conventional cytotoxic treatment is already being pursued to a rather large extent. Apart from this, the addition of an angiogenesis inhibiting agent following VDA administration conceivably could induce ‘synergistic’ antiangiogenic activity leading to a complete growth inhibition and a subsequent state of dormancy of the ‘centrally killed’ tumour mass. This observation has meanwhile indeed been made in preclinical models (Siemann and Shi, 2004). In addition, the combination of low-dose chemotherapy following the administration of a VDA could also be considered. Here one could think of the concept of metronomic chemotherapy.

Exploring a combination of VDAs and epidermal growth factor receptor (EGFR) inhibitors theoretically could also be an interesting approach; here one could speculate that tumour cells in the viable rim will become apoptotic and die when being deprived of their growth-stimulating factors such as EGF.

Effectiveness of combination therapies often depends on the sequence of administration. First increasing the vascular permeability (within minutes) allowing accumulation of a cytotoxic agent in the tumour and then inducing a shutdown of blood flow (within hours) could probably kill large amounts of tumour cells. However, as certain chemotherapeutics have their own vascular side effects (arterial thrombosis following administration of cisplatin), the alternative sequence of first administering the VDA followed by the administration of a cytotoxic drug could also be considered. Irrespective of the sequence administered, these combination therapies should be carefully chosen and closely monitored.

From a theoretical perspective, radiation therapy probably must precede VDA administration because optimal blood flow and oxygen radical formation might be necessary for an optimal synergistic effect. Preclinical models have meanwhile shown such a synergistic effect. Finally, and considered as a very specific example, in tumours of the extremities, one could think of isolated limb perfusion with VDAs after clamping; Biologically active concentrations of VDAs could probably be achieved with only limited systemic exposure, thus leading to optimal and selective tumour cell killing while preserving the heart, central nervous system and other potential organs at risk. Monitoring antivascular effects with either angiography, DCE–MRI or PET would be challenging.

In conclusion, VDAs are a new and promising class of targeted anticancer agents. Although their safety currently is the major focus of research, results that will show anticancer activity are likely to follow soon.

Their unique mechanism of action merits thorough and extensive exploration, both as single agent as well as in combination with other treatment modalities. If these studies are performed adequately, with a close observation of toxicity, it is to be expected that in the years to come a clear picture of their role in anticancer treatment can be established.

## Figures and Tables

**Table 1:** Currently published phase-I studies

**Drug**	**Company**	**Treatment schedule**	**Dose range**	**DLT^a^**	**References**
CA4P	OXiGEN	d 1,8,15 q4w d 1–5 q3w d 1 q3w	5–114 mg m^−2^ i.v. 6–75 mg m^−2^ i.v. 18–90 mg m^−2^ i.v.	Bowel ischaemia, tumour pain, vagal syncope, motor neuropathy, reversible ataxia, cardiac ischaemia, dyspnoe	Rustin *et al* Stevenson *et al* Dowlati and Cooney *et al*
AVE8062	Sanofi Aventis	d 1,8,15 q 4	4.5–40 mg m^−2^ i.v.	Transient myocardial ischaemia, asymp hypotension, transient neurological symptoms	Tolcher *et al*
ZD6126	Astra Zeneca	weekly d 1 q3w d 1 q3w	5–28 mg m^−2^ i.v. 5–40 mg m^−2^ i.v. 5–112 mg m^−2^ i.v.	Myocardial infarction, pulm. embolus, LVEF decrease, fatigue	Beerepoot *et al* LoRusso *et al* Gadgeel *et al*
ABT-751	Abbott	q.d. 7 days q3w b.i.d 7 days q3w	200–300 mg po 125–175 mg po	Ileus, constipation, abdominal pain, neuropathy, fatigue	Hande *et al*
MN-029	MediciNova	3-weekly	4–180 mg m^−2^ i.v.	Reversible cardiac ischaemia	Ricart *et al*
TZT- 1027	Daichi Pharmaceuticals	d 1,8 q3w d1,8 q3w+carbo AUC 4–5 d 1 q3w	1.35–2.7 mg m^−2^ i.v. 1.6–2.0 mg m^−2^ i.v. 1.35–3.0 mg m^−2^ i.v.	Neutropenia, pain infusion arm, peripheral neuropathy fatigue, ileus	de Jonge *et al* Greystoke *et al* Schoffski *et al*
DMXAA	Antisoma	Weekly d 1 q3w	6–4900 mg m^−2^ i.v. 6–4900 mg m^−2^ i.v.	Reversible urinary incontinence, visual disturbances, anxiety	Rustin *et al* Jameson *et al*

DLT=dose-limiting toxicity; i.v.=intravenously. Ref. =References.

## References

[bib1] Baguley BC, Zhuang L, Kestell P (1997) Increased plasma serotonin following treatment with flavone-8-acetic acid, 5,6-dimethylxanthenone-4-acetic acid, vinblastine, and colchicines: relation to vascular effects. Oncol Res 9: 55–60 9167186

[bib2] Beauregard DA, Thelwall PE, Chaplin DJ, Hill SA, Adams GE, Brindle KM (1998) Magnetic resonance imaging and spectroscopy of combretastatin A4 prodrug-induced disruption of tumour perfusion and energetic status. Br J Cancer 77: 1761–1767 9667644 10.1038/bjc.1998.294 PMC2150333

[bib3] Beerepoot LV, Radema SA, Witteveen EO, Thomas T, Wheeler C, Kempin S, Voest E (2006) Phase I clinical evaluation of weekly administration of the novel vascular-targeting agent, ZD6126, in patients with solid tumors. J Clin Oncol 24: 1491–1498 16574998 10.1200/JCO.2005.02.7458

[bib4] Bilenker JH, Flaherty KT, Rosen M, Davis L, Gallagher M, Stevenson JP, Sun W, Vaughn D, Giantonio B, Zimmer R, Schnall M, O'Dwyer PJ (2005) Phase I trial of combretastatin a-4 phosphate with carboplatin. Clin Cancer Res 11: 1527–1533 15746056 10.1158/1078-0432.CCR-04-1434

[bib5] Chaplin DJ, Dougherty GJ (1999) Tumour vasculature as a target for cancer therapy. Br J Cancer 80(Suppl 1): 57–64 10466764

[bib6] Ching LM, Cao Z, Kieda C, Zwain S, Jameson MB, Baguley BC (2002) Induction of endothelial cell apoptosis by the antivascular agent 5,6-Dimethylxanthenone-4-acetic acid. Br J Cancer 86: 1937–1942 12085190 10.1038/sj.bjc.6600368 PMC2375421

[bib7] Cooney MM, Radivoyevitch T, Dowlati A, Overmoyer B, Levitan N, Robertson K, Levine SL, DeCaro K, Buchter C, Taylor A, Stambler BS, Remick SC (2004) Cardiovascular safety profile of combretastatin a4 phosphate in a single-dose phase I study in patients with advanced cancer. Clin Cancer Res 10: 96–100 14734457 10.1158/1078-0432.ccr-0364-3

[bib8] Corbett TH, Bissery MC, Wozniak A, Plowman J, Polin L, Tapazoglou E, Dieckman J, Valeriote F (1986) Activity of flavone acetic acid (NSC-347512) against solid tumors of mice. Invest New Drugs 4: 207–220 3546183 10.1007/BF00179586

[bib9] Dark GG, Hill SA, Prise VE, Tozer GM, Pettit GR, Chaplin DJ (1997) Combretastatin A-4, an agent that displays potent and selective toxicity toward tumour vasculature. Cancer Res 57: 1829–1834 9157969

[bib10] Davis PD, Dougherty GJ, Blakey DC, Galbraith SM, Tozer GM, Holder AL, Naylor MA, Nolan J, Stratford MR, Chaplin DJ, Hill SA (2002) ZD6126: a novel vascular-targeting agent that causes selective destruction of tumor vasculature. Cancer Res 62: 7247–7253 12499266

[bib11] DeJonge MJ, van der Gaast A, Planting AS, van Doorn L, Lems A, Boot I, Wanders J, Satomi M, Verweij J (2005) Phase I and pharmacokinetic study of the dolastatin 10 analogue TZT-1027, given on days 1 and 8 of a 3-week cycle in patients with advanced solid tumors. Clin Cancer Res 10: 3806–3813 10.1158/1078-0432.CCR-04-1937 15897580

[bib12] Demers B, Vrignaud P, Bissery M (2006) *In vivo* synergy combining oxaliplatin with AVE8062, a vascular-disrupting agent. J Clin Oncol 24(suppl): 607s (abstract 13074)

[bib13] Denekamp J (1982) Endothelial cell proliferation as a novel approach to targeting tumour therapy. Br J Cancer 45: 136–139 7059456 10.1038/bjc.1982.16 PMC2010961

[bib14] Dowlati A, Robertson K, Cooney M, Petros WP, Stratford M, Jesberger J, Rafie N, Overmoyer B, Makkar V, Stambler B, Taylor A, Waas J, Lewin JS, McCrae KR, Remick SC (2002) A phase I pharmacokinetic and translational study of the novel vascular targeting agent combretastatin A-4 phosphate on a single-dose intravenous schedule in patients with advanced cancer. Cancer Res 62: 3408–3416 12067983

[bib15] Escudier B, Eisen T, Stadler WM, Szczylik C, Oudard S, Siebels M, Negrier S, Chevreau C, Solska E, Desai AA, Rolland F, Demkow T, Hutson TE, Gore M, Freeman S, Schwartz B, Shan M, Simantov R, Bukowski RM (2007) Sorafenib in advanced clear-cell renal-cell carcinoma. New Engl J Med 356: 125–134 17215530 10.1056/NEJMoa060655

[bib16] Ferrara N, Gerber HP, LeCouter J (2003) The biology of VEGF and its receptors. Nat Med 9: 669–676 12778165 10.1038/nm0603-669

[bib17] Folkman J (1971) Tumor angiogenesis: therapeutic implications. N Engl J Med 285: 1182–1186 4938153 10.1056/NEJM197111182852108

[bib18] Gabra H (2006) Phase II study of DMXAA combined with carboplatin and paclitaxel in recurrent ovarian cancer. J Clin Oncol 24(Suppl): 263s (abstract 5032)

[bib19] Gadgeel SM, LoRusso P, Wozniak AJ, Wheeler C (2002) A dose escalation study of the novel vascular targeting agent ZD6126 in patients with solid tumours. J Clin Oncol 21(Suppl): 110a (abstract 438)

[bib20] Galbraith SM, Maxwell RJ, Lodge MA (2003) Combretastatin A4 phosphate has tumor antivascular activity in rat and man as demonstrated by dynamic magnetic resonance imaging. J Clin Oncol 21: 2831–2842 12807936 10.1200/JCO.2003.05.187

[bib21] Greystoke A, Blagden S, Thomas AL, Scott E, Attard G, Molife R, Vidal L, Pacey S, Sarkar D, Jenner A, De-Bono JS, Steward W (2006) A phase I study of intravenous TZT-1027 administered on day 1 and day 8 of a three-weekly cycle in combination with carboplatin given on day 1 alone in patients with advanced solid tumours. Ann Oncol 8: 1309–1313 10.1093/annonc/mdl097 16728482

[bib22] Hande KR, Hagey A, Berlin J, Cai Y, Meek K, Kobayashi H, Lockhart AC, Medina D, Sosman J, Gordon GB, Rothenberg ML (2006) The pharmacokinetics and safety of ABT-751, a novel orally bioavailable sulfonamide antimitotic agent: results of a phase I study. Clin Cancer Res 9: 2834–2840 10.1158/1078-0432.CCR-05-2159 16675578

[bib23] Heeckeren van WJ (2006) Promise of new vascular-disrupting agents balanced with cardiac toxicity: is it time for the oncologists to get to know their cardiologists? J Clin Oncol 24: 1485–1488 16574996 10.1200/JCO.2005.04.8801

[bib24] Hori K, Saito S, Kubota K (2002) A novel combretastatin A-4 derivative, AC7700, strongly stanches tumour blood flow and inhibits growth of tumours developing in various tissues and organs. Br J Cancer 86: 1604–1614 12085211 10.1038/sj.bjc.6600296 PMC2746587

[bib25] Hurwitz H, Fehrenbacher L, Novotny W, Cartwright T, Hainsworth J, Heim W, Berlin J, Baron A, Griffing S, Holmgren E, Ferrara N, Fyfe G, Rogers B, Ross R, Kabbinavar F (2004) Bevacizumab plus irinotecan, fluorouracil, and leucovorin for metastatic colorectal cancer. N Engl J Med 350: 2335–2342 15175435 10.1056/NEJMoa032691

[bib26] Jameson MB, Thompson PI, Baguley BC, Evans BD, Harvey VJ, Porter DJ, McCrystal MR, Small M, Bellenger K, Gumbrell L, Halbert GW, Kestell P (2003) Clinical aspects of a phase I trial of 5,6-dimethylxanthenone-4-acetic acid (DMXAA), a novel antivascular agent. Br J Cancer 88: 1844–1850 12799625 10.1038/sj.bjc.6600992 PMC2741109

[bib27] Kakolyris S, Fox S, Koukourakis M, Giatromanolaki A, Brown N, Leek RD, Taylor M, Leigh IM, Gatter KC, Harris AL (2000) Relationship of vascular maturation in breast cancer blood vessels to vascular density and metastasis, assesed by expression of a novel basement membrane component, LH39. Br J Cancer 82: 844–851 10732757 10.1054/bjoc.1999.1010 PMC2374391

[bib28] Kerbel R, Folkman J (2002) Clinical translation of angiogenesis inhibitors. Nat Rev Cancer 2: 727–739 12360276 10.1038/nrc905

[bib29] Koehne C, Bajetta E, Lin E, van Cutsem E, Hecht J, Douillard J, Morre M, Germond C, Laurent D (2006) Results of an interim analysis of a multinational randomized, double-blind, phase III study in patients (pts) with previously treated metastatic colorectal cancer (mCRC) receiving FOLFOX4 and PTK787/ZK 222584 (PTK/ZK) or placebo (CONFIRM 2). J Clin Oncol 24(Suppl): 148s (abstract 3508)

[bib30] Konerding MA, Fait E, Gaumann A (2001) 3D microvascular architecture of pre-cancerous lesions and invasive carcinomas of the colon. Br J Cancer 84: 1354–1362 11355947 10.1054/bjoc.2001.1809 PMC2363651

[bib31] LoRusso S, Gadgeel SM, Wozniak AJ (2002) A phase I dose escalation trial of ZD6126, a novel vascular targeting agent, in patients with cancer refractory to other treatments. Proc AACR-NCI-EORTC (abstract 36)

[bib32] McGown AT, Fox BW (1989) Structural and biochemical comparison of the anti-mitotic agents colchicine, combretastatin A4 and amphethinile. Anti Cancer Drud Des 3: 249–254 2930627

[bib33] McKeage M (2006) Phase Ib/II study of DMXAA combined with carboplatin and paclitaxel in non-small cell lung cancer (NSCLC). J Clin Oncol 24(Suppl): 389s (abstract 7102)

[bib34] Motzer RJ, Hutson TE, Tomzak P, Michaelson MD, Bukowski RM, Rixe O, Oudard S, Negrier M, Szczylik C, Kim ST, Chen I, Bycott PW, Baum CM, Figlin RA (2007) Sunitinib *vs* Interferon Alfa in metastatic renal cell carcinoma. N Engl J Med 356: 115–124 17215529 10.1056/NEJMoa065044

[bib35] Ozawa Y, Sugi NH, Nagasu T, Owa T, Wantanabe T, Koyanagi N, Yoshino H, Kitoh K, Yoshimatsu K (2001) E7070, a novel sulfonamide agent with potent antitumor activity *in vitro* and *in vivo*. Eur J Cancer 37: 2275–2282 11677118 10.1016/s0959-8049(01)00275-1

[bib36] Ramaswamy B, Elias AD, Kelbick NT, Dodley A, Morrow M, Hauger M, Allen J, Phoades C, Kendra K, Chen HX, Eckhardt SG, Shapiro CL (2006) Phase II trial of bevacizumab in combination with weekly docetaxel in metastatic breast cancer patients. Clin Cancer Res 10: 3124–3129 10.1158/1078-0432.CCR-05-2603 16707611

[bib37] Ricart AD, Cooney M, Sarantopoulos J, Brell J, Locke KW, Gammans RE, Medina G, Zambito A, Tolcher W, Remick SC (2006) A phase I pharmacokinetic (PK) and pharmacodynamic (PD) study of MN-029, a novel vascular disrupting agent (VDA), in patients with advanced solid tumors. J Clin Oncol 24(Suppl): 144s (abstract 3096)

[bib38] Rustin GJ, Galbraith SM, Anderson H, Stratford M, Folkes LK, Sena L, Gumbrell L, Price PM (2003a) Phase I clinical trial of weekly combretastatin A4 phosphate: clinical and pharmacokinetic results. J Clin Oncol 21: 2815–2822 12807934 10.1200/JCO.2003.05.185

[bib39] Rustin GJS, Bradley C, Galbraith S, Stratford M, Loadman P, Waller S, Bellenger K, Gumbrell L, Folkes L, Halbert G (2003b) 5,6-dimethylxanthenone-4-acetic acid (DMXAA), a novel antivascular agent: phase I clinical and pharmacokinetic study. Br J Cancer 88: 1160–1167 12698178 10.1038/sj.bjc.6600885 PMC2747563

[bib40] Sandler A, Gray R, Perry MC, Brahmer J, Schiller JH, Dowlati A, Lillenbaum R, Johnson DH (2006) Paclitaxel–Carboplatin alone or with bevacizumab for non-small cell lung cancer. N Engl J Med 355: 2542–2550 17167137 10.1056/NEJMoa061884

[bib41] Schoffski P, Thate B, Beutel G, Bolte O, Otto D, Hofmann M, Ganser A, Jenner A, Cherverton P, Wanders J, Oguma T, Atsumi R, Satomi M (2004) Phase I and pharmacokinetic study of TZT-1027, a novel synthetic dolastatin 10 derivative, administered as a 1-h intravenous infusion every 3 weeks in patients with advanced refractory cancer. Ann Oncol 4: 671–679 10.1093/annonc/mdh141 15033678

[bib42] Shi W, Siemann DW (2005) Preclinical studies of the novel vascular disrupting agent MN-029. Anticancer Res 25: 3899–3904 16309177

[bib43] Siemann DW, Bibby MC, Dark GG, Dicker AP, Eskens FA, Horsman MR, Marme D, Lorusso PM (2005) Differentiation and definition of vascular-targeted therapies. Clin Cancer Res 11: 416–420 15701823

[bib44] Siemann DW, Shi W (2004) Efficacy of combined antiangiogenic and vascular disrupting agents in treatment of solid tumors. Int J Rad Oncol 60: 1233–1240 10.1016/j.ijrobp.2004.08.002 15519796

[bib45] Stevenson JP, Rosen M, Sun W, Gallagher M, Haller DG, Vaughn D, Giantonio B, Zimmer R, Petros WP, Stratford M, Chaplin D, Young SL, Schnall M, O'Dwyer PJ (2003) Phase I trial of the antivasular agent combretastatin A4 phosphate on a 5-day schedule to patients with cancer: magnetic resonance imaging evidence for altered blood flow. J Clin Oncol 21: 4428–4438 14645433 10.1200/JCO.2003.12.986

[bib46] Thomsen LL, Ching LM, Zhuang L, Gavin JB, Baguley BC (1991) Tumor-dependent increased plasma nitrate concentrations as an indication of the antitumor effect of flavone-8-acetic acid and analogues in mice. Cancer Res 51: 77–81 1988109

[bib47] Tolcher AW, Forero L, Celio P, Hammond LA, Patnaik A, Hill M, Verat-Follet C, Haacke M, Besenval M, Rowinsky EK (2003) Phase I, pharmacokinetic, and DCE–MRI correlative study of AVE8062A, an antivascular combretastatin analogue, administered weekly for 3 weeks every 28 days. J Clin Oncol 22(Suppl): 208 (abstract 834)

[bib48] Tozer GM, Lewis S, Michalowski A, Aber V (1990) The relationship between regional variations in blood flow and histology in a transplanted rat fibrosarcoma. Br J Cancer 61: 250–257 2310676 10.1038/bjc.1990.46 PMC1971396

[bib49] Tozer GM, Prise VE, Wilson J, Locke RJ, Vojnovic B, Stratford MR, Dennis MF, Chaplin DJ (1999) Combretastatin A-4-phosphate as a tumor vascular targeting agent: early effects in tumors and normal tissues. Cancer Res 59: 1626–1634 10197639

[bib50] Vincent L, Kermani P, Young LM, Cheng J, Zhang F, Shido K, Lam G, Bompais-Vincent H, Zhu Z, Hicklin DJ, Bohlen P, Chaplin DJ, May C, Rafii S (2005) Combretastatin A4 phosphate induces rapid regression of tumor neovessels and growth through interference with vascular endothelial-cadherin signaling. J Clin Investigation 115: 2992–3006 10.1172/JCI24586 PMC1253622 16224539

[bib51] Yang JC, Haworth L, Sherry RM, Hwu P, Schwartzentruber DJ, Topalian SL, Steinberg SM, Chen HX, Rosenberg SA (2003) A randomized trial of bevacizumab, an anti-vascular endothelial growth factor antibody, for metastatic renal cancer. N Engl J Med 349: 427–434 12890841 10.1056/NEJMoa021491 PMC2275324

